# The activity of therapeutic molecular cluster Ag5 is dependent on oxygen level and HIF-1 mediated signalling

**DOI:** 10.1016/j.redox.2024.103326

**Published:** 2024-08-22

**Authors:** Sophie A. Twigger, Blanca Dominguez, Vanesa Porto, Lina Hacker, Anthony J. Chalmers, Ross Breckenridge, Martin Treder, Adam C. Sedgwick, Fernando Dominguez, Ester M. Hammond

**Affiliations:** a Department of Oncology, University of Oxford, Oxford, OX3 7DQ, UK; b Department of physiology and CIMUS Universidade de Santiago de Compostela, Spain; c School of Cancer Sciences, University of Glasgow, UK; d Arjuna Therapeutics, Milladoiro, Spain; e Department of Chemistry, King's College London, London, SE1 1DB, UK

**Keywords:** Hypoxia, Redox, Ag5, HIF-1, Radiation

## Abstract

Regions of hypoxia occur in most solid tumours and are known to significantly impact therapy response and patient prognosis. Ag5 is a recently reported silver molecular cluster which inhibits both glutathione and thioredoxin signalling therefore limiting cellular antioxidant capacity. Ag5 treatment significantly reduces cell viability in a range of cancer cell lines with little to no impact on non-transformed cells. Characterisation of redox homeostasis in hypoxia demonstrated an increase in reactive oxygen species and glutathione albeit with different kinetics. Significant Ag5-mediated loss of viability was observed in a range of hypoxic conditions which mimic the tumour microenvironment however, this effect was reduced compared to normoxic conditions. Reduced sensitivity to Ag5 in hypoxia was attributed to HIF-1 mediated signalling to reduce PDH via PDK1/3 activity and changes in mitochondrial oxygen availability. Importantly, the addition of Ag5 significantly increased radiation-induced cell death in hypoxic conditions associated with radioresistance. Together, these data demonstrate Ag5 is a potent and cancer specific agent which could be used effectively in combination with radiotherapy.

## Highlights

• Characterisation of redox in a range of hypoxic conditions.
• Ag5 is a therapeutic molecular cluster toxic to cancer cells and not normal cells.
• Sensitivity to Ag5 is rescued by loss of HIF-mediated signalling to PDK1/3.

## Graphical abstract

**Figure:**
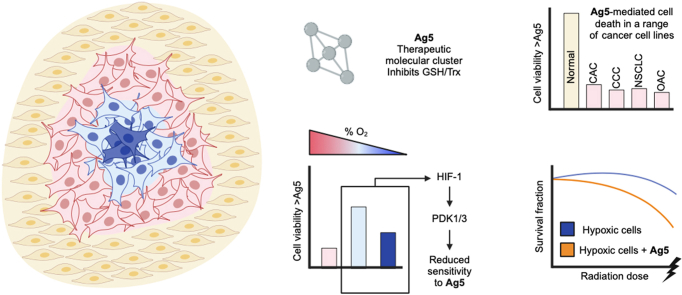
Gradients of oxygen exist within solid tumours, including well-oxygenated (pink) areas and those that are increasingly hypoxic (blue). Ag5 inhibits both GSH and Trx therefore impacting the main cellular anti-oxidant pathways. Ag5 exposure has little to no effect on normal cells while leading to loss of viability in a range of cancer cell lines. Sensitivity to Ag5 in hypoxia can be rescued through loss of HIF-1a. Hypoxic cells are radiosensitised by the addition of Ag5. CAC – colorectal adenocarcinoma, CCC- clear cell carcinoma, NSCLC – non small cell lung carcinoma, OAC- oesophageal adenocarcinoma. Figure created with BioRender.Com, license number WK25R9XLMQ.

## 1 Introduction

Cancer cells can be characterised by an imbalance in reduction-oxidation (redox) homeostasis and specifically elevated levels of reactive oxygen species (ROS) [1]. High levels of free radical species have been attributed to increased metabolic activity, oncogene activation (e.g. K-Ras or c-Myc mutations) and disruption of key antioxidant signalling pathways [2,3]. The redox state provides a targetable difference between normal and cancer cells that can be therapeutically exploited.

Most solid tumours have some degree of hypoxia, due to abnormal vasculature and high metabolic demand [4,5]. The impact of hypoxia on ROS levels is controversial with some studies demonstrating increased ROS production in normal and malignant cells under hypoxia [[6], [7], [8], [9]], whilst others reporting the opposite [[10], [11], [12], [13]], suggesting a complex dependence on cell line, experimental conditions and specific ROS subtype. Where described, increased ROS levels under hypoxic conditions are thought to originate from complex I/III of the mitochondrial electron transport chain, the NADPH oxidase enzyme family and iron release from the Fenton reaction [[14], [15], [16]]. Hypoxia-induced ROS have been shown to contribute to the stabilisation of the hypoxia-inducible transcription factor, HIF-1, a heterodimeric protein complex composed of an oxygen-sensitive alpha subunit (HIF-1α) and a constitutively expressed HIF-1β subunit [17]. Activation of HIF-1 leads to numerous downstream responses including upregulation of anti-oxidant signalling [18,19]. More recently, ROS levels in hypoxia have been shown to contribute to transcriptional stress through the accumulation of DNA:RNA hybrids, R-loops [20].

The two main antioxidant mechanisms within the cell are the glutathione (GSH) and thioredoxin (Trx) pathways, both of which are regulated by the signalling molecule nicotinamide adenine dinucleotide (NADPH) which maintains them in a reduced state [21]. An upregulation of cellular GSH contributes to redox homeostasis in hypoxic conditions, balancing out increases in both ROS and reactive nitrogen species (RNS) [7]. In addition, the uptake of l-cysteine, which is essential for GSH synthesis, is reported to increase in hypoxia via upregulation of dedicated transporter systems (X_c_^−^ system) [7].

Redox-dependent agents such as doxorubicin have been described, where conversion to a semiquinone moiety by cellular reductases produces high levels of ROS in the cell [22]. Furthermore, a number of agents have been designed to target cellular antioxidant pathways. For example, buthionine sulfoximine (BSO), ebselen and auranofin, increase ROS levels beyond the threshold that cancer cells can tolerate, thereby inducing cell death [17,23,24]. As an example, BSO induces apoptosis by inhibiting γGCS, the first enzyme in GSH biosynthesis, decreasing the levels of intracellular GSH and leaving the cell susceptible to oxidative stress [23]. However, pre-clinical testing of these drugs revealed off target toxicity or limited efficacy which was attributed to a failure to inhibit all the antioxidant systems [25].

More recently, a therapeutic molecular cluster composed of five silver atoms (Ag5) was reported to inhibit both the GSH and Trx antioxidant pathways [26]. Using ROS species as substrates, Ag5 catalyses the irreversible oxidation of thiol groups present on cysteine residues within GSH/Trx, leaving them unable to reduce ROS/RNS and alleviate cellular stress. The Ag5-dependent accumulation of ROS in the cell leads to apoptosis [26]. In addition, the use of molecular clusters, such as Ag5, presents an attractive therapeutic strategy due to their small size, compared to most therapeutics, allowing the penetration of poorly vascularised regions such as those that lead to tumour hypoxia.

Therapy resistance in hypoxia is well-characterised, with many of the underpinning mechanisms known, however the response of hypoxic cells to ROS-dependent therapies remains unclear partly due to the incomplete understanding of how hypoxia affects redox homeostasis [5,27]. Here, we investigate the impact of oxygen levels on redox homeostasis and the efficacy of a ROS-dependent therapeutic, Ag5, in these physiological conditions.

## 2 Results

### 2.1 Ag5 leads to loss of viability in cancer but not normal cells

**Fig. 1:**
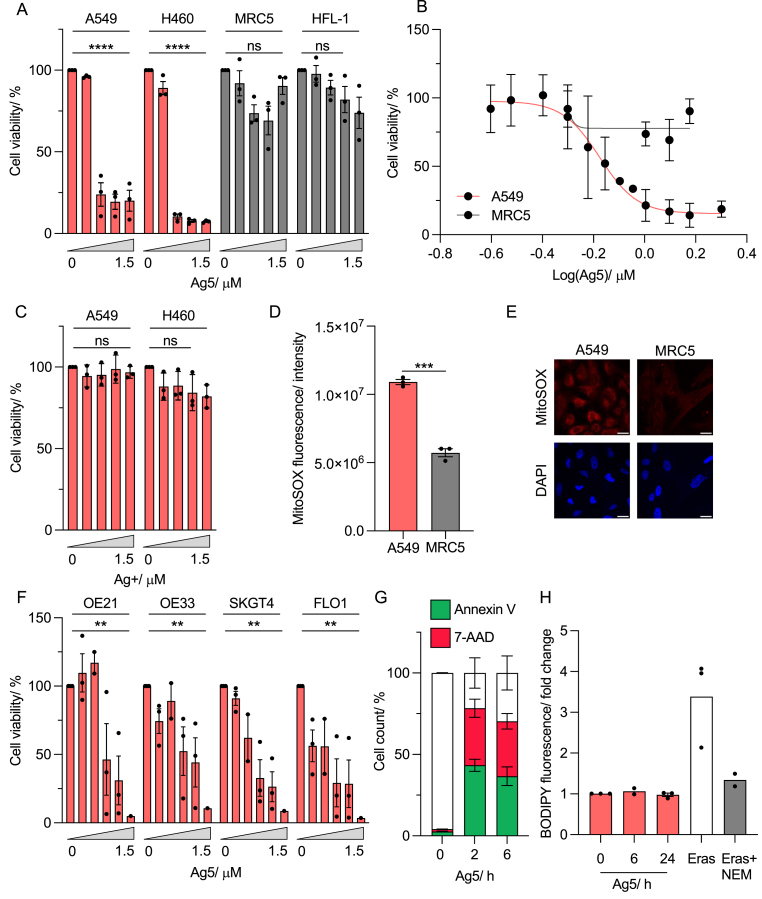
**Ag5 is toxic to cancer cells but not non-transformed cells.** **A**. Lung cancer lines (A549, H460) and non-transformed lung lines (MRC5, HFL-1) were treated with Ag5 (0, 0.5, 1, 1.25, 1.5 μM) for 1 h and analysed via MTT 20 h later. **B**. A549 and MRC5 cells were treated with Ag5 (0, 0.25, 0.3, 0.4, 0.5, 0.6, 0.7, 0.8, 0.9, 1, 1.25, 1.5, 2 μM) for 1 h and analysed via MTT 20 h later. **C**. A549 and H460 cells were treated with the control compound, Ag+ (0, 0.5, 1, 1.25, 1.5 μM) for 1 h, followed by an MTT assay 20 h later. **D**. Levels of ROS (MitoSOX) were determined in untreated A549 and MRC5 cells. **E**. Representative images from part D. Scale bar = 20 μm **F**. A panel of oesophageal cancer cell lines (OE21, OE33, SKGT4, FLO1) were treated with Ag5 (0, 0.5, 1, 1.25, 1.5 μM) for 1 h and an MTT assay was carried out after 20 h **G**. A549 cells were treated with Ag5 (1 μM) for 1 h followed by annexin-V and 7-AAD assay for apoptosis after 2 and 6 h **H.** A549 cells were treated with Ag5 (1 μM) for 1 h, followed by a lipid peroxidation assay 6 and 24 h after drug treatment. Erastin (10 μM) was used as a positive control and NEM (200 μM) to verify that the fluorescence recorded was due to lipid peroxidation. Data shown in A, B, C, D, E, F and G are n = 3. Data shown in part H is n = 3 except for the Erastin + NEM condition which is n = 2. Black dots on the graphs shown represent biological repeats (each of which was carried out in triplicate, except for the IF). Data presented as mean + SEM. Statistical testing was done using a two-way ANOVA test for the cell viability assays or a *t*-test for the lipid peroxidation assay. **p < 0.01, ***p < 0.001, ****p < 0.0001, ns = non-significant.

The response to Ag5 in the lung adenocarcinoma cell lines, A549 and H460 was compared to non-transformed cell lines, MRC5 and HFL-1 (both lung fibroblast). Using an MTT assay, a significant loss of viability was determined in response to Ag5 in both A549 and H460 cells in a dose dependent manner. In contrast, little to no effect on viability was observed in the normal cells (Fig. 1A). A549 cells were exposed to additional doses of Ag5 in order to calculate the IC50, which was found to be approximately 0.67 μM. In contrast it was not possible to determine the IC50 for Ag5 using the same doses in the non-transformed MRC5 cell line (Fig. 1B). To confirm that the observed toxicity was independent of the presence of silver ions, we used Ag + as a control compound. Cell survival was not impacted by exposure to Ag+ in A549 or H460 cells (Fig. 1C). As expected, the levels of ROS (mitochondrial) were found to be significantly higher in the A549 cells compared to the non-transformed MRC5 cells, supporting the reported mechanism of action for Ag5. (Fig. 1D and E). To demonstrate that Ag5 activity is not restricted to lung cancer cell lines, we tested a panel of oesophageal cancer cell lines. Here, we also observed a dose-dependent loss of viability in response to Ag5 (Fig. 1F). Notably, there did not appear to be a difference in sensitivity to Ag5 across the cell lines tested, suggesting sensitivity is not restricted to the presence of specific mutations (Table S1). As previously reported, Ag5-mediated cell death was found to be apoptotic (Fig. 1G) [26]. We hypothesised that other cell death pathways may also be involved. Specifically, we asked if Ag5 led to ferroptosis as this is a ROS-dependent form of cell death resulting from lipid peroxidation [28]. However, no significant increase in lipid peroxidation was found in response to Ag5 in the tested conditions, suggesting increased ferroptosis is not a relevant cell death mechanism (Fig. 1H). Overall, our results show a dose-dependent toxicity of Ag5, specifically in cancer cells.

### 2.2 Hypoxia leads to increases in ROS and GSH levels

**Fig. 2:**
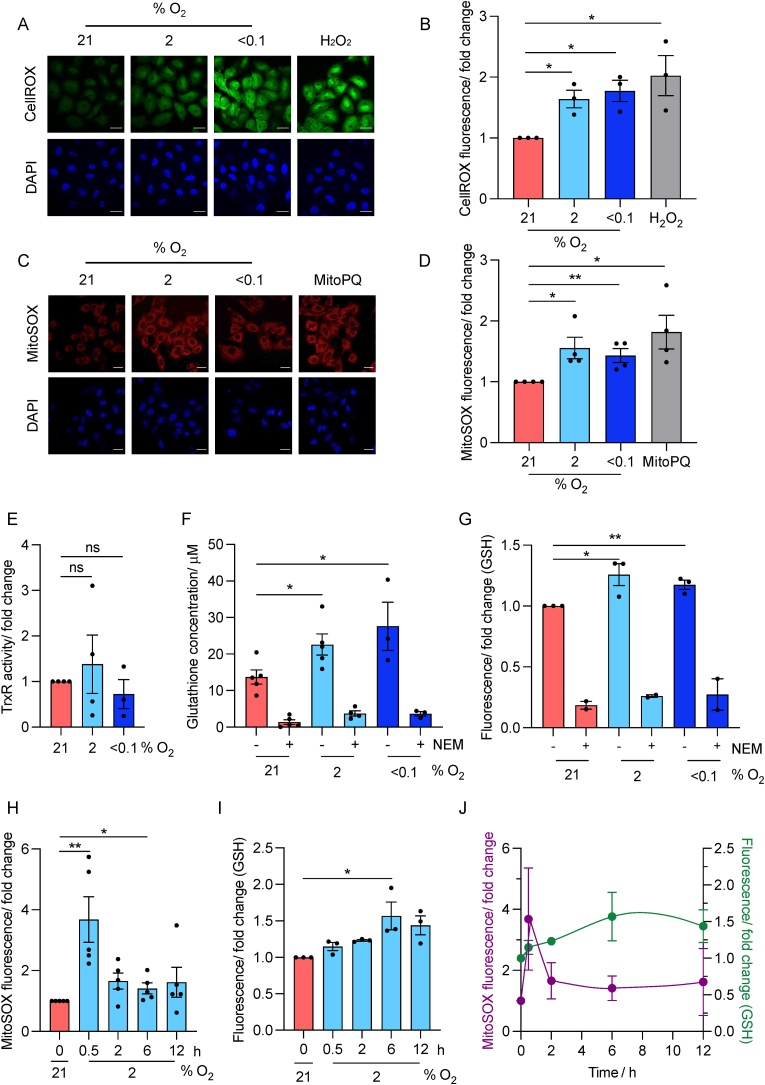
**Changes to redox in hypoxic conditions.** **A**. A549 cells were exposed to 21, 2 and < 0.1 % O_2_ (6 h) followed by staining for total ROS (CellROX). H_2_O_2_ (25 μM, 3 h) was used as a positive control to induce ROS. Representative images are shown. Scale bar = 12 μm **B**., Quantification of total ROS fold change in A549 cells stained using the CellROX assay in 21, 2, <0.1 % O_2_ (6 h). H_2_O_2_ (25 μM) was added as a control that induces ROS. **C**. A549 cells were exposed to 21, 2 and < 0.1 % O_2_ (6 h) followed by staining for mitochondrial ROS (MitoSOX). MitoPQ (10 μM, 6 h) was used as positive control. Representative images are shown. Scale bar = 12 μm. **D**. Quantification of mitochondrial ROS fold change in A549 cells stained using the MitoSOX assay in 21, 2 and < 0.1 % O_2_ (6 h). MitoPQ (10 μM) was added as a control that induces mitochondrial ROS. **E**. The fold change in TrxR activity was determined in A549 cells exposed to 21, 2 or <0.1 % O_2_ for 6 h **F**. A549 cells were exposed to the O_2_ levels shown (6 h) and GSH levels were determined (GSH GLO assay). In each case NEM (200 μM, 6 h) was included to verify that the luminescence recorded was due to GSH. **G**. A549 cells were exposed to the O_2_ levels shown (6 h) and GSH levels determined using FL-1. In each case NEM (200 μM, 6 h) was included to verify that the fluorescence recorded was due to GSH. **H**. A549 cells were exposed to 2 % O_2_ (0, 0.5, 2, 6, 12 h) followed by staining for mitochondrial ROS (MitoSOX). **I**. A549 cells were exposed to 2 % O_2_ (0, 0.5, 2, 6, 12 h) followed by determination of GSH levels using FL-1. **J.** The data shown in parts H and I are plotted together to illustrate the differential kinetics of hypoxia induced mitochondrial ROS levels (MitoSOX) and GSH levels (FL-1) in A549 cells exposed to 2 % O_2_ (0, 0.5, 2, 6, 12 h). Data shown in A, B, C, D, E, F, G, H and I are n = 3/4. Black dots on the graphs shown represent biological repeats (each of which was carried out in triplicate, except for the IF). Data presented as mean + SEM. Statistical testing was done using an unpaired *t*-test. *p < 0.05, **p < 0.01, ns = non-significant.

The specific ratio of O_2_/H_2_O_2_ has been described as critical for the action of Ag5. H_2_O_2_ binds to Ag5 with a lower adsorption energy than it binds to O_2_, however, the catalytic activity of Ag5 is increased when bound to H_2_O_2_ in comparison to O_2_ [26]. Therefore, before further testing of Ag5, we characterised the changes in redox in physiologically relevant conditions, using a combination of assays for ROS and the main cellular antioxidants, Trx and GSH. To begin, we measured oxidative stress using probes for both total cellular ROS (Fig. 2A and B) and mitochondrial ROS (superoxide, O^2•-^) (Fig. 2C and D). In both cases a significant increase in ROS was determined in the hypoxic conditions tested (2 or <0.1 % O_2_). Next, we measured Trx levels and found no significant change in either of the hypoxic conditions tested (2 or <0.1 % O_2_) (Fig. 2E). However, in agreement with previous reports, we found that GSH levels increased in response to hypoxia (Fig. 2F) [7,[29], [30], [31]]. N-Ethylmaleimide (NEM) was included as a negative control to confirm the cellular GSH level measurements, as NEM contains an alkene functional group that reacts with thiols present on the cysteines within GSH. To investigate GSH levels further, we synthesised a thiol responsive fluorescent probe (FL-1), that utilised the thiol reactive 2,4-DNB protecting group to mask the fluorophore 3-*O*-methylfluorescein and quench the fluorescence emission [25]. Thus, upon reaction with GSH, the 2,4-DNB unit is removed, and the highly fluorescent fluorescein moiety is released allowing the measurement of changes in GSH concentrations. GSH-mediated changes were confirmed in solution along with thiol selectivity and in cells using N-acetylcysteine (NAC) and NEM (Figs. S1A–E). Using FL-1, we again determined a significant increase in GSH in hypoxic conditions (2 and < 0.1 % O_2_) (Fig. 2G). Importantly, all the assays carried out were carried out without including reoxygenation and therefore reflect redox status in the hypoxic conditions described at the time of testing. The increase of both ROS and GSH at the 6 h time point in hypoxic conditions prompted us to explore further the kinetics of these changes. A549 cells were exposed to hypoxia (2 % O_2_) and mitochondrial ROS and GSH levels determined over a 12-h period. As previously, both ROS and GSH were significantly increased after exposure to 2 % O_2_ for 6 h, however these data also demonstrated that ROS levels peaked after just 30 min (Fig. 2H). In contrast, GSH levels did not peak until 6 h exposure to hypoxia (Fig. 2I) confirming differential kinetics of GSH and ROS with the antioxidant response lagging behind the initial burst in oxidative stress (Fig. 2J). The observed changes in redox likely underpin differential sensitivities to cancer therapeutics including Ag5 in hypoxia. However, the exact impact of these changes specifically on Ag5 sensitivity remains unclear. If the increase in ROS was more biologically significant, the sensitivity to Ag5 would be predicted to increase, whilst if the increase in GSH was more relevant, the sensitivity would be expected to decrease.

### 2.3 Hypoxic cells are less sensitive to Ag5

**Fig. 3:**
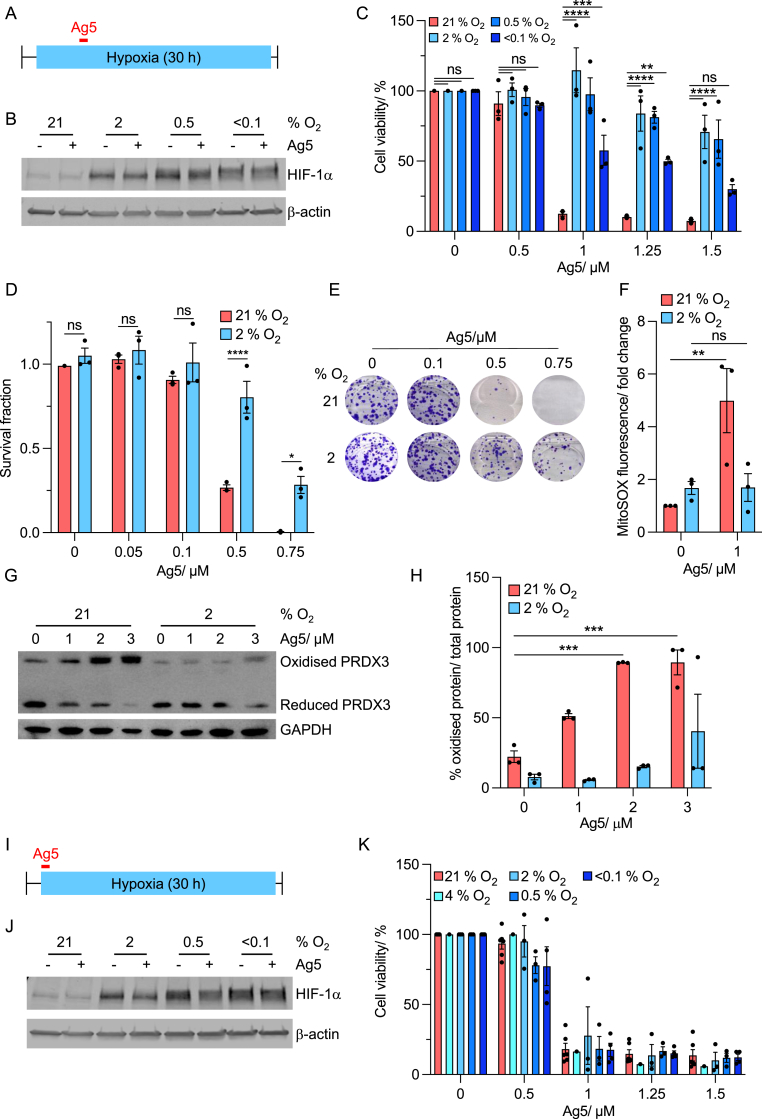
**Hypoxic cells are less sensitive to Ag5.** **A**. Schematic representation of cell viability assays with Ag5 with pre-exposure to hypoxic conditions. Cells were placed in hypoxia for 6 h and then treated with Ag5 for 1 h **B**. A549 cells were exposed to 21, 2, 0.5 and < 0.1 % O_2_ (6 h) before treatment with or without Ag5 (1 μM) for 1 h. After 20 h samples were harvested for western blotting of HIF-1α and β-actin as a loading control. **C**. A549 cells were exposed to 21, 2, 0.5 and < 0.1 % O_2_ (6 h) and then treated with Ag5 (0, 0.5, 1, 1.25, 1.5 μM) for 1 h, followed by an MTT assay 20 h later. **D**. A549 cells were exposed to 21, 2, 0.5 and < 0.1 % O_2_ (6 h) and then treated with Ag5 (0, 0.5, 1, 1.25, 1.5 μM) for 1 h, followed by a colony survival assay. **E**. Representative images of the colonies formed in part D. **F**. A549 cells were exposed to 21 or 2 % O2 (4 h) followed by Ag5 treatment (5 min) and staining for mitochondrial ROS (MitoSOX). **G**. A549 cells were exposed to 21 and 2 % O_2_ (4 h) and then treated with Ag5 (0, 1, 2, 3 μM) for 15 min, followed by western blotting for PRDX3, with GAPDH as a loading control. **H**. Quantification of the percentage of oxidised/reduced PRDX3 in A549 cells after exposure to 21 and 2 % O_2_ (4 h) followed by Ag5 treatment (15 min) and western blotting. **I**. Schematic representation of cell viability assays with Ag5 with no pre-exposure to hypoxic conditions. Cells were exposed to Ag5 for 1 h **J.** A549 cells were treated with Ag5 (1 μM) and then immediately exposed to 21, 2, 0.5 and < 0.1 % O_2_. Media was changed after 1 h and western blotting was carried out 20 h later for HIF-1α with β-actin as a loading control. **K**. A549 cells were treated with Ag5 (0, 0.5, 1, 1.25, 1.5 μM) and then immediately exposed to 21, 2, 0.5 and < 0.1 % O_2_. Media was changed after 1 h and an MTT assay was carried out 20 h later. Data shown in C, D, E, F, G, H and K are n = 3/4. Black dots on the graphs shown represent biological repeats (each of which was carried out in triplicate). Data presented as mean + SEM. Statistical testing was done using a two-way ANOVA test with each bar compared to the 21 % O_2_ counterpart. *p ≤ 0.05, **p < 0.01, ***p < 0.001, ****p < 0.0001, ns = non-significant.

Having demonstrated that redox homeostasis is changed by hypoxia and therefore likely to impact sensitivity to Ag5, we exposed A549 cells to hypoxia (<0.1–2 % O_2_) followed by treatment with Ag5 (Fig. 3A). As expected, HIF-1α was stabilised in response to all the hypoxic conditions tested (2, 0.5 and < 0.1 % O_2_) and this was not impacted by the addition of Ag5 (Fig. 3B). In each of the conditions, A549 cells were less sensitive to Ag5 in hypoxia compared to normoxia (Fig. 3C). Notably, despite no significant difference in the response to Ag5 in 2 and 0.5 % O_2_, the cells treated at <0.1 % O_2_ were more sensitive to Ag5 compared to the other hypoxic conditions. The biological response to hypoxia is oxygen dependent and it is likely that accumulation of replication stress and subsequent DNA damage response signalling specifically at <0.1 % O_2_ may explain the sensitivity to Ag5 compared to milder levels of hypoxia (Fig. S2A) [20]. To further verify these findings, we carried out a colony survival assay in A549 cells treated with Ag5 in either normoxia or hypoxia (2 % O_2_). Supporting our previous findings, cells treated in hypoxia were significantly less sensitive to Ag5 compared to those in normoxia (Fig. 3D and E). Importantly, this oxygen-dependent sensitivity to Ag5 was also observed in H460 cells demonstrating that it was not a cell line dependent effect (Figs. S2B and C).

Together, these data suggest that Ag5 is less active in hypoxic conditions. Ag5 has been shown to lead to increased ROS as a result of loss of GSH/Trx activity, therefore we measured ROS (mitochondrial) after exposure to Ag5 in both normoxic and hypoxic (2 % O_2_) conditions. As expected, a significant increase in ROS were determined in normoxia after Ag5 treatment but not in hypoxia (Fig. 3F). The oxidation/reduction ratio of the mitochondrial antioxidant enzyme, thioredoxin-dependent peroxide reductase (PRDX3), has been shown previously to be a primary mediator of Ag5 action [26]. Therefore, we pre-exposed A549 and H460 cells to hypoxia (2 % O_2_), followed by Ag5 treatment (as in Fig. 3A) and determined the oxidation status of PRDX3 (Fig. 3G, H, S2D, E). As expected, in normoxia, the level of oxidised PRDX3 increased and the level of reduced PRDX3 decreased in an Ag5-dependent manner, confirming that the reduction/oxidation ratio PRDX3 could be used as a marker of Ag5 activity. However, the ratio of oxidised/reduced PRDX3 was reduced in hypoxia compared to normoxia. These data indicate that Ag5 is less active in hypoxic conditions, correlating with the reduced toxicity observed.

To investigate further, we exposed cells treated with Ag5 to hypoxia, but without the pre-exposure to hypoxia (Fig. 3I). Our hypothesis was that due to the rapid increase in ROS observed previously and relatively slow increase in GSH activity (Fig. 2H and I), sensitivity to Ag5 could be unchanged using this scheduling. As expected, we saw no difference in sensitivity to Ag5 in either A549 or H460 cells in normoxia versus hypoxia (Fig. 3J, K, S2F, G). Together, these data suggest that the increase in GSH levels observed in hypoxia have more of an impact on sensitivity to Ag5 than the changes in ROS levels.

### 2.4 Sensitivity to Ag5 in hypoxia is rescued by loss of HIF-1α

**Fig. 4:**
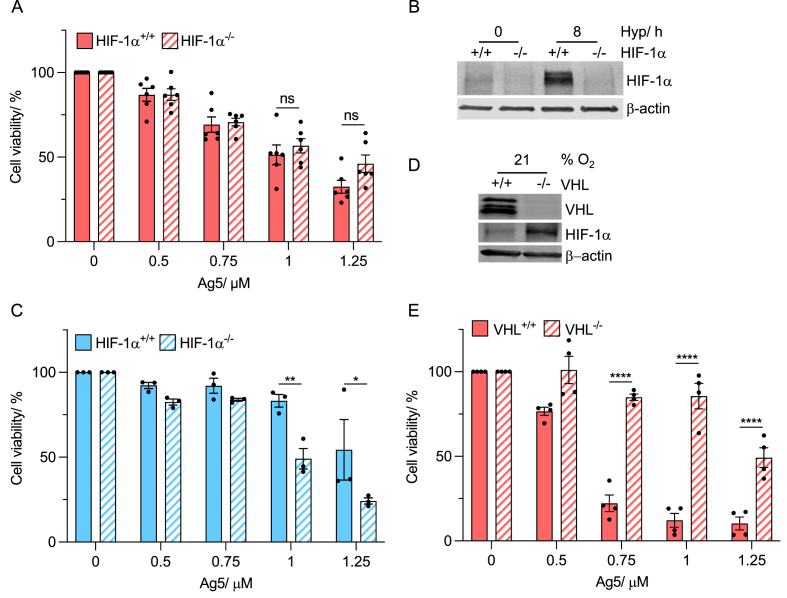
**Sensitivity to Ag5 is HIF-1α dependent.** **A**. RKO and RKO^HIF−1α−/−^ cells were treated with Ag5 (0, 0.5, 0.75, 1, 1.25 μM) for 1 h in 21 % O_2_ followed by an MTT assay 20 h later. **B**. RKO and RKO^Hif−1α−/−^ cells were exposed to 21 % and <0.1 % O_2_ (8 h) followed by western blotting for HIF-1α, with β-actin as a loading control. **C**. RKO and RKO^HIF−1α−/−^ cells were exposed to 2 % O_2_ (6 h), followed by treatment with Ag5 (0, 0.5, 0.75, 1, 1.25 μM) for 1 h and then an MTT assay 20 h later **D**. Untreated RCC4 and RCC4^VHL−/−^ cells were western blotted for VHL, HIF-1⍺ and a loading control (β-actin). **E**. RCC4 and RCC4^VHL−/−^ cells were treated with Ag5 (0, 0.5, 0.75, 1, 1.25 μM) for 1 h in 21 % O_2_ followed by an MTT assay 20 h later. Data shown in A, C and E are n = 3. Black dots on the graphs shown represent biological repeats (each of which was carried out in triplicate). Data presented as mean + SEM. Statistical testing was done using a two-way ANOVA test. *p < 0.05, **p < 0.01, ****p < 0.0001, ns = non-significant.

Given the necessity of pre-exposure to hypoxia to decrease response to Ag5, we hypothesised that HIF-1 could play a role in determining sensitivity to Ag5. We used two models to investigate the role of HIF-1 in the reduced sensitivity seen in hypoxia to Ag5. First, we used the genetically matched colorectal carcinoma cell lines (RKO^HIF−1α+/+^ and RKO^HIF−1α−/−^). In normoxia, no significant difference in sensitivity to Ag5 was found between the two cell lines and as expected neither stabilised HIF-1α (Fig. 4A and B). In contrast, when Ag5 was added in hypoxic conditions (2 % O_2_), the RKO^HIF−1α−/−^ cells were significantly more sensitive compared to RKO^HIF−1α+/+^ and HIF-1α was stabilised (Fig. 4B and C). As expected, RKO^HIF−1a+/+^ cells showed higher Ag5 sensitivity in normoxia compared to hypoxia (2 % O_2_) (Fig. S3A). These data confirm that HIF-1α plays a role in sensitivity to Ag5. To further investigate, we employed the RCC4^VHL+/+^ and RCC4^VHL−/−^ cell lines as models of pseudo-hypoxia, as HIF-1α is stabilised in normoxic conditions in the VHL null cells (Fig. 4D) [32]. Cells lacking VHL were significantly less sensitive to Ag5 compared to the VHL proficient cells in normoxia (Fig. 4E), further supporting the role of HIF-1α in mediating Ag5 sensitivity.

### 2.5 Impact of HIF-1α signalling on redox

**Fig. 5:**
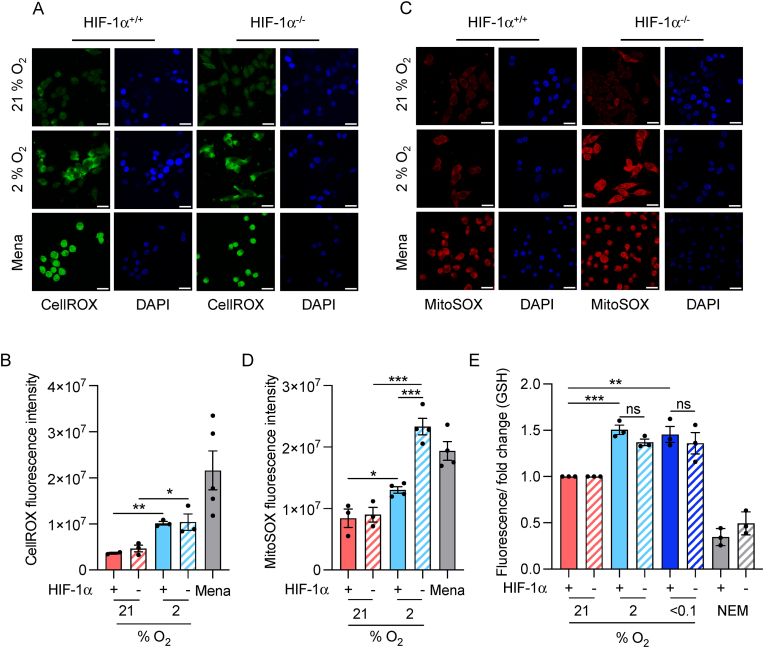
**The role of HIF-1 in redox.** **A.** RKO and RKO^HIF−1α−/−^ cells were exposed to 21 or 2 % O_2_ (6 h) followed by staining for total ROS (CellROX). Menadione (100 μM, 6 h) was used as a positive control to induce ROS. Scale bar = 20 μm **B.** Quantification of total ROS in RKO and RKO^HIF−1α−/−^ cells stained using the CellROX assay in 21 and 2 % O_2_ (6 h). As a control, menadione (100 μM) was added to induce ROS. **C.** RKO and RKO^HIF−1α−/−^ cells were exposed to 21 or 2 % O_2_ (6 h) followed by staining for mitochondrial ROS (MitoSOX). Menadione (100 μM, 6 h) was used as a positive control to induce ROS. Scale bar = 20 μm. **D.** Quantification of mitochondrial ROS in RKO and RKO^HIF−1α−/−^ cells stained using the MitoSOX assay in 21 and 2 % O_2_ (6 h). As a control, menadione (100 μM) was added to induce ROS. **E.** RKO and RKO^Hif−1α−/−^ cells were exposed to the O_2_ levels shown (6 h) and GSH levels determined (FL-1). In each case NEM (200 μm, 6 h) was also included to verify that the fluorescence recorded was due to GSH. Data shown in A, B, C, D and E are n = 3. Black dots on the graphs shown represent biological repeats (each of which was carried out in triplicate, except for the IF). Data presented as mean + SEM. Statistical testing was done using an unpaired *t*-test. *p < 0.05, **p < 0.01, ***p < 0.001 ns= non-significant.

Next, as both ROS and GSH levels increased in hypoxic conditions, we investigated if this was a HIF-dependent effect. To explore the impact of HIF-1 on ROS levels, we exposed RKO^HIF−1α+/+^ and RKO^HIF−1α−/−^ cells to normoxia and hypoxia (2 % O_2_) and measured total cellular and mitochondrial ROS levels. Supporting our earlier finding that ROS levels increase in hypoxia, we saw a significant increase in fluorescence associated with CellROX (total cellular ROS) when the cells were exposed to 2 % O_2_. However, no significant difference was observed between the cell lines indicating that total ROS levels were not altered in a HIF-1 dependent manner (Fig. 5A and B). As previously, an increase in MitoSOX was observed in RKO cells however, the loss of HIF-1α was found to lead to a further increase (Fig. 5C and D). In order to test if HIF-1 played a significant role in the hypoxic increase of GSH, we measured GSH levels in the RKO^HIF−1α+/+^ and RKO^HIF−1α−/−^ cells in normoxic and hypoxic conditions (2 and < 0.1 % O_2_). As previously and using both assays, GSH levels increased in hypoxia but, were not significantly altered by loss of HIF-1 α (Fig. 5 E–S 3B). Together, these data support the hypothesis that loss of HIF-1α restores sensitivity to Ag5 by increasing mitochondrial ROS, which is expected to enhance Ag5 activity, and likely by also reducing HIF-1-mediated signalling to genes with anti-oxidant functions [18,19].

### 2.6 Sensitivity to Ag5 is restored in hypoxia by loss of PDK1/3

**Fig. 6:**
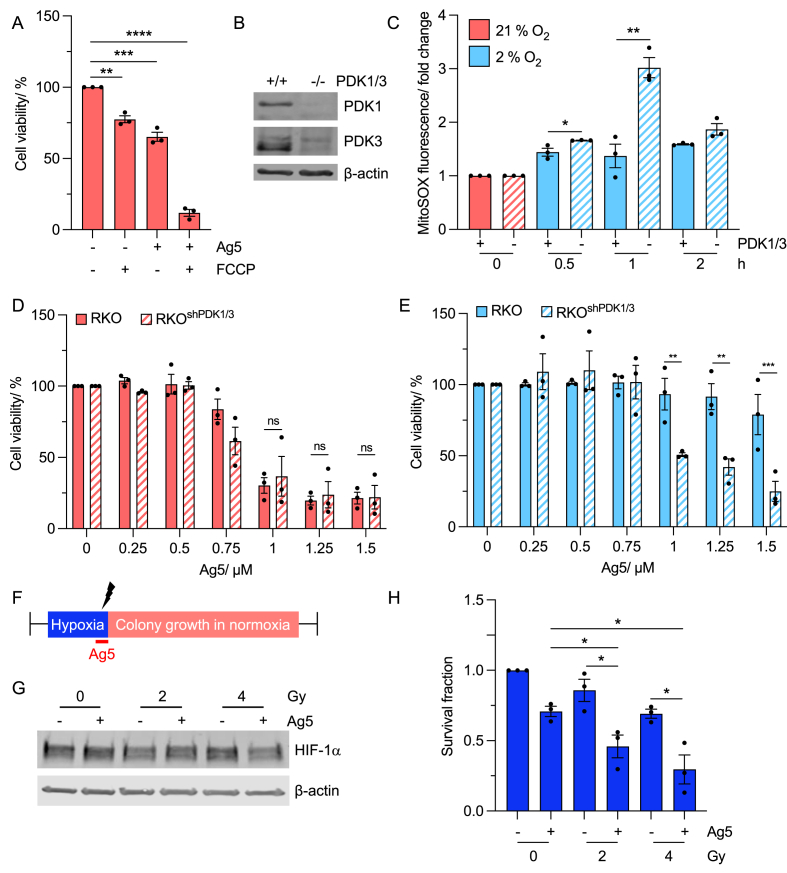
**Sensitivity to Ag5 in hypoxia can be restored by altering cellular redox.** **A.** A549 cells were either untreated or treated with FCCP (6 h), Ag5 (1 h) or both FCCP (6 h) and Ag5 (1 h) followed by an MTT assay 20 h later. **B.** Levels of PDK1, PDK3 and a loading control (β-actin) are shown in untreated RKO and RKO^shPDK1/3^ cells. **C.** RKO and RKO^shPDK1/3^ cells were exposed to 2 % O_2_ (0, 0.5, 1, 2, 3, 6 h) followed by staining for mitochondrial ROS (MitoSOX). **D.** RKO and RKO^shPDK1/3^ cells were treated with Ag5 (0, 0.25, 0.5, 0.75, 1, 1.25 μM) for 1 h in normoxia (21 % O_2_) followed by an MTT assay 20 h later. **E.** RKO and RKO^shPDK1/3^ cells were exposed to hypoxia (2 % O_2_) (6 h), followed by treatment with Ag5 (0, 0.5, 0.75, 1, 1.25 μM) for 1 h followed by an MTT assay 20 h later. **F.** Schematic representation of the experimental set up for combining Ag5 with radiation in hypoxia (not to scale). Importantly, radiation (indicated with black lightning strike) was delivered in hypoxic conditions i.e. without reoxygenation. Colonies were then allowed to form in normoxic (21 % O_2_) conditions. **G.** A549 cells were exposed to <0.1 % O_2_ (4 h), followed by treatment with Ag5 (1 μM) for 1 h. During Ag5 treatment, cells were irradiated (0, 2, 4 Gy) in hypoxic conditions. Cells were harvested after the 1 h treatment and western blotted for HIF-1α, with β-actin as a loading control. **H.** A549 cells were treated as in part E followed by return to normoxic conditions (21 % O_2_), and colony survival assay. Data shown in A, C, D, E and H are n = 3. Black dots on the graphs shown represent biological repeats (each of which was carried out in triplicate). Data presented as mean + SEM. Statistical testing was done using a two-way ANOVA test for cell viability and unpaired *t*-test for colony survival. *p < 0.05, **p < 0.01, ***p < 0.001, ****p < 0.0001 ns= non-significant.

To support our hypothesis that changes in mitochondrial ROS impact sensitivity to Ag5, we treated A549 cells with a combination of Ag5 and FCCP, which leads to an increase in ROS through uncoupling of mitochondrial oxidative phosphorylation [33]. As predicted the combination of Ag5 and FCCP had a greater impact on cell viability compared to either agent alone (Fig. 6A). In response to hypoxia, the glycolytic pathways are upregulated, due to increased expression of glucose transporters e.g. Glut1, which are HIF-1 targets [[34], [35], [36]]. In contrast, oxidative phosphorylation (OXPHOS) is inhibited in hypoxia via HIF-1 mediated increases in pyruvate dehydrogenase kinase (PDK) expression which prevents pyruvate dehydrogenase (PDH) fuelling the tricarboxylic acid (TCA) cycle with pyruvate (Fig. S3C) [32]. By blocking PDH, the amount of oxygen available in the mitochondria is increased, therefore altering the ratio of O_2_/H_2_O_2_. Both PDK1/3 are well characterised targets of HIF-1α [[37], [38], [39]]. We predicted that in the absence of PDK1/3, the ratio of O_2_/H_2_O_2_ in hypoxia would be more similar to that in normoxia and sensitivity to Ag5 would be restored. In order to test this hypothesis, we used RKO cells with reduced expression of PDK1 and PDK3 [32]. As expected, mitochondrial ROS levels increased more in response to hypoxia when PDK1/3 levels were depleted (Fig. 6B and C). We exposed RKO and RKO^shPDK1/3^ cells to 21 or 2 % O_2_, treated them with Ag5 (as in Fig. 3A) and determined cell viability via MTT assay. As expected, the RKO cells were less sensitive to Ag5 at 2 % O_2_ compared to 21 % O_2_. In normoxia, there was no significant difference in sensitivity to Ag5 between the two cell lines (Fig. 6D). However, the RKO^shPDK1/3^ cells were significantly more sensitive to Ag5 in hypoxia (2 % O_2_), compared to the wildtype RKO cells (Fig. 6E). These data demonstrate that sensitivity to Ag5 in hypoxia can be restored through loss of PDK1/3 and more broadly through combination with agents which lead to increased mitochondrial ROS levels.

### 2.7 Ag5 increases the radiosensitivity of hypoxic cells

Despite the reduced sensitivity to Ag5 in hypoxia, or conditions where HIF-1 is expressed, Ag5 is a promising anti-cancer agent due to the lack of toxicity observed in non-transformed cells (Fig. 1A). Radiation in the form of radiotherapy leads to the generation of hydroxyl (^•^OH) radicals from the hydrolysis of water which cause DNA damage and cell death [40]. Therefore, we asked if the combination of Ag5 and radiation would be effective. Importantly, we investigated the impact of Ag5 on radiosensitivity in hypoxic conditions as hypoxia is a major mechanism of radiation resistance [41]. A549 cells were exposed to radiobiological hypoxia (<0.1 % O_2_) and treated with both Ag5 and radiation (0, 2, 4 Gy) and a colony survival assay carried out (Fig. 6F). As expected, the cells irradiated in hypoxic (<0.1 % O_2_) conditions were significantly more radioresistant compared to those treated in normoxic conditions (Fig. S3D) and HIF-1α was stabilised in all conditions (Fig. 6G). The combination of Ag5 and radiation had a greater effect on cell survival compared to either agent alone (Fig. 6H). Together, these data suggest that the combination of Ag5 and radiotherapy would be efficacious and does not increase hypoxia-induced radioresistance.

## 3 Discussion

The therapeutic molecular cluster Ag5 leads to significant loss of viability in a range of cancer cell lines but not non-transformed cells therefore making it an attractive anti-cancer agent. Here, we investigated how the activity of Ag5 was impacted by conditions which reflect the physiological levels of oxygen commonly found in solid tumours. In all of the hypoxic conditions tested, cells were less sensitive to Ag5 compared to normoxic (21 % O_2_) conditions, but notably hypoxic cancer cells were still more sensitive to Ag5 compared to the non-transformed cells. Normal cells experiencing the levels of hypoxia found in tumours and tested here are rare within the normal body providing an exploitable therapeutic window. Importantly, the combination of radiation and Ag5 significantly increased cell death in hypoxia compared to radiation alone. The decreased sensitivity to Ag5 observed in hypoxia was found to be HIF-1α dependent and more specifically dependent on the HIF-1 target genes PDK1/3. Our data supports the hypothesis that loss of PDK1/3 in hypoxic conditions relieves the inhibitory effect of PDH on oxygen consumption and therefore increases available oxygen and ROS in the mitochondria which is the site of Ag5 action. Ag5 is known to be exquisitely sensitive to the ratio of O_2_/H_2_O_2_ [26].

As part of this study, we also provide a detailed characterisation of the redox environment in hypoxic conditions including changes in ROS (total and mitochondrial) and the major antioxidant systems. Our study demonstrates that, in the absence of reoxygenation, hypoxia leads to an increase in ROS and GSH levels, supported by previous literature [7,28,42,43]. Importantly, we demonstrate that the kinetics of the increase in ROS and GSH differ with ROS levels peaking quickly (30 min) and GSH lagging behind. The increase in mitochondrial ROS was found to be HIF-1α dependent. In contrast, total cellular ROS and GSH levels were not impacted by HIF-1α activity in our hands. Notably, a previous study found GSH synthesis to be induced by chemotherapy in a HIF-dependent manner in triple negative breast cancer (TNBC), indicating that there is likely a dependence on experimental conditions [44].

In summary, Ag5 is an exciting addition to the class of redox dependent agents with anti-cancer activity. The high potency of Ag5 in cancer cells is likely underpinned by targeting both GSH and Trx pathways therefore preventing cancer cells circumventing reduced antioxidant capacity. Our study highlights the importance of testing anti-cancer agents in conditions which mimic the hypoxic tumour microenvironment as Ag5 is less toxic in these conditions. However, Ag5 remains a promising anti-cancer approach as it shows selective activity in cancer compared to normal cells and combines well with radiation.

## 4 Methods

### 4.1 Cells, cell culture and reagents

Human non-small cell lung carcinoma cells A549 (ATCC) and H460 (Prof. Geoff Higgins, University of Oxford) were grown in DMEM. Human oesophageal cancer OE21, OE33, FLO1 and SK-GT-4 cells (Dr Ricky Sharma, University of Oxford) were grown in DMEM (OE21 and FLO1) and RPMI (OE33 and SK-GT-4). Human colorectal RKO and RKO^HIF−1α−/−^ cancer cells were grown in DMEM [45]. RKO^shPDK1/3^ cells (Prof. Nicholas Denko, The Ohio State University) were also grown in DMEM [37]. Human renal clear carcinoma RCC4 and RCC4^VHL−/−^ cancer cell lines (Prof. Sir Peter Ratcliffe, University of Oxford) were grown in DMEM. Human foetal lung fibroblast cell lines MRC5 and HFL-1 (Prof. Geoff Higgins, University of Oxford), were grown in DMEM and nutrient mixture F12 HAM media, respectively. All media was supplemented with 10 % FBS and 1 % P/S and cells were cultured in humified incubators at 37 °C with 5 % CO_2_. All cell lines were verified to be negative for mycoplasma using a Lonza MycoAlert® mycoplasma detection assay (LT07-318, Lonza). Ag5/Ag+ was provided by Arjuna Therapeutics. A stock solution of Ag5 was prepared in water and stored at room temperature, protected from light. The stock solution was sonicated for 30–60 min prior to every use and diluted in serum-free DMEM before adding to cells. Cells were exposed to Ag5/Ag + for 1 h in serum free media, which was then replaced with complete media.

### 4.2 Ag5 treatment

Cells were treated with Ag5 in FBS free media for 1 h, except for the PRDX3 assay in which treatment was 15 min, media was then changed to complete serum containing media. In hypoxic conditions, FBS free media and complete serum media were equilibrated to the desired O_2_ concentration before use.

### 4.3 MTT assay

Cells were seeded in flat bottomed 96 well plates and allowed to adhere for 2–4 h (37 °C, 5 % CO_2_), before being exposed to a range of O_2_ conditions. Each treatment condition was carried out in triplicate. After 20 h, MTT reagent (0.5 mg/mL, Invitrogen) was added for 3 h (37 °C) with the plate protected from light. The plates were then removed from their respective oxygen environment and the MTT containing media was discarded. DMSO (100 μL) was added to each well and the plate was incubated in the dark for 15 min (37 °C) before measuring absorbance (570 nm) (POLARstar plate reader, BMG LabTech). Cell viability was calculated as a percentage in comparison to the untreated control. The IC50 was calculated using non-linear regression.

### 4.4 Annexin-V assay

After treatment, cells were washed twice with PBS and then stained with Annexin V-FITC (1:10, 15 min, BD biosciences, 550,911) and 7-AAD (5 μM, 15 min, BD biosciences, 555,816), following the manufacturer's instructions. Images were obtained using an Olympus IX51 microscope equipped with an Olympus DP72 camera and CellSens Imaging Software and processed with ImageJ.

### 4.5 Lipid peroxidation assay

After drug treatment, the media was removed and cells were incubated with BODIPY™ 581/591 lipid peroxidation sensor (2 μM, Invitrogen, D3861) in PBS for 30 min at 37 °C, away from light. Ferrostatin (5 μM) was used as a positive control and was added at the same time as seeding. Cells were then trypsinised in the dark and the pellet resuspended in FACs buffer (PBS, 2 % FBS, 0.5 mM EDTA). Samples were then analysed on a CytoFLEX flow cytometer (Beckman Coulter) using CytExpert software. Analysis was carried out for a cell count of 10,000 events for each treatment condition in triplicate. FloJo software (BD Biosciences) was used to analyse the data.

### 4.6 ROS assays

Intracellular and mitochondrial ROS levels were determined using the CellROX™ Green (Thermo Fisher Scientific, C10444) and MitoSOX™ Red (Thermo Fisher Scientific, M36008), respectively. After drug/hypoxia treatment cells were incubated with either 10 μM CellROX™ Green (30 min) or 5 μM MitoSOX™ Red (10 min) at 37 °C in the dark. H_2_O_2_ (25 μM, 3 h, Sigma-Aldrich), MitoPQ (10 μM, 6 h) and menadione (100 μM, 6 h, Merck) were used as controls. After staining, cells were fixed using 4 % PFA (10 min), washed three times with PBS and mounted with ProLong™ Diamond Antifade Mountant with DAPI (Invitrogen). Microscopy imaging was carried out using a Zeiss 780 or 710 confocal Microscope (Carl Zeiss Ag, Jena, Germany) at 60× magnification and at least 100 cells were counted per condition for quantification.

### 4.7 GSH assays

The GSH-Glo assay was carried out according to the manufacturer's instructions (Promega, V6911). Each treatment condition was performed in triplicate. Cells were seeded (4000/well) in white walled, flat bottomed 96 well plates and allowed to adhere overnight. Plates were then placed in the stated oxygen tension and were either untreated or pre-treated with NEM (200 μM) for 6 h. A standard curve was generated at 21 % O_2_.

Synthesis of the novel GSH probe (FL-1) is described in the supplementary information. To assay GSH using FL-1, cells were seeded (4000/well) in flat bottomed 96 well plates and allowed to adhere overnight. Plates were then placed in the stated oxygen tensions and were either untreated or pre-treated with NEM (200 μM) for 6 h. FL-1 (10 μM) was then added to each well and the contents of the well was mixed. After 1 h, the fluorescence was measured (POLARstar plate reader, BMG LabTech).

### 4.8 Trx assay

The TrxR assay was carried out, to investigate Trx levels, according to the manufacturer's instructions (AbCam, ab83463). Each treatment condition was performed in triplicate. Cells were seeded in glass dishes and allowed to adhere overnight. Plates were then placed in the required oxygen tension for 6 h. Cells were then harvested and assayed for Trx using the TrxR assay kit, colorimetric (AbCam, ab83463).

### 4.9 Hypoxia treatment

Cells were exposed to hypoxia at the stated oxygen tensions using a Bactron II anaerobic chamber (Shel labs) for <0.1 % O_2_ and an M35 hypoxia workstation (Don Whitley) for 0.5–4 % O_2_. Glass dishes were used for hypoxia experiments, except when plastic plates were required (MTT, GSH or colony assays). Oxygen concentrations were periodically validated using anaerobic oxygen indicator strips (Thermo Fisher) and an OxyLite (Oxford Optronix).

### 4.10 Immunoblotting

Cells were harvested in UTB lysis buffer (9 M Urea, 75 mM Tris-HCl pH 7.5, 0.15 M β-mercaptoethanol) and briefly sonicated. Proteins were separated on a 4–20 % polyacrylamide gel (Bio-Rad) and transferred onto a nitrocellulose membrane (Bio-Rad). The Odyssey Infrared Imaging System (LI-COR Biosciences) was used. Antibodies used were HIF-1α (BDBiosciences, 610,959), β-Actin (Santa Cruz, 69,879), VHL (Cell Signalling, 68,547), Glut-1 (Abcam, ab14683), Gapdh (Protein tech, 60,004-1-1 g), PDK1 (AbCam, ab110025), PDK3 (AbCam, 154,549), p53-S15 (Cell Signalling, 92,845) and p53 (Santa Cruz, sc-126).

### 4.11 Colony survival assay

Cells were seeded at the appropriate density, depending on the treatment condition, in 6-well plates to give a total volume of 2 mL per well. Seeding was calculated to give a plating efficiency above 50 % for the untreated condition. Each treatment condition was performed in triplicate. After treatment (Ag5/IR/hypoxia) colonies were allowed to grow in a humified incubator (37 °C, 5 % CO_2_) for 14 days. Once the colonies had formed a sufficient size (>50 cells), the media was removed and they were stained with crystal violet (0.5 % w/v in 50 % MeOH and 20 % EtOH) for 1 h, before washing with water. Colonies were counted using a manual cell counter (Stuart Scientific). The survival fraction was calculated by no. Of colonies counted/no. Of cells seeded × PE, where PE is the plating efficiency of the untreated control (no. Of colonies counted/no. Of cells seeded).

### 4.12 PRDX3 assay

Cells were seeded in plastic dishes and allowed to adhere overnight, prior to drug/hypoxia treatment. The media was then replaced with 80 mM methyl methanethiosulfonate (MMTS) (Sigma, 64,306) enriched media to prevent oxidation of reduced thiols. The dishes were washed 2 x ice cold PBS, followed by addition of 75 μL RIPA buffer (50 mM Tris, pH 7.5, 150 mM NaCl, 1 % (v/v) Triton-X100, 0.1 % (w/v) SDS, 0.5 % (w/v) sodium deoxycholate) supplemented with 1 mM sodium orthovanadate, 1 mM PMSF, 5 mM NaF, 1x Protease inhibitor cocktail and 80 mM MMTS. The cells were scraped into the buffer after 20 min incubation on ice. The samples were sonicated (30 % amplitude, 1 s on, 1 s off x 10) and then centrifuged (14,000 rpm, 20 min, 4 °C). Samples were mixed with loading buffer (250 mM Tris-Cl (pH 6.8), 8 % SDS, 0.1 % bromophenol blue, 40 % (v/v) glycerol, H_2_O) (non-oxidising), heated (96 °C, 5 min) and electrophoresed on a 15 % acrylamide gel (250 V, 50 A). The gel was transferred (1 h) onto a PVDF membrane and blocked with milk powder (1 h), washed 3 x TNT (0.1 % TWEEN) and incubated with antibody (PRDX3, Abcam, ab129206) (4 °C, overnight). The membrane was then washed 3 x TNT (0.1 % TWEEN), incubated with 2° antibody (1 h, rt), followed by washing 3 x TNT (0.1 % TWEEN). ECL reagent was then added, and the gels were visualised using a ChemiDoc Imaging System (BioRad). The membranes were then blotted for GAPDH (Sigma, MAB374), followed by a HRP 2° antibody and then visualised using a ChemiDoc.

### 4.13 Radiation treatment

Irradiation was carried out using γ-rays from a Cs-137 irradiator (GSM: GSR D1) at a dose rate of 1.7 Gy/min, at rt. For irradiating experiments in hypoxia, cells were transferred to airtight Perspex plastic boxes to allow for transportation to and from the irradiator [32].

### 4.14 Statistical analysis

GraphPad Prism software (GraphPad software Inc.) was used to carry out all statistical analysis, including two-way ANOVA tests and unpaired t-tests. Data is presented as mean + SEM. Statistical testing is described in each figure legend. *p < 0.05, **p < 0.01, ***p < 0.001, ****p < 0.0001, ns = not significant.

## CRediT authorship contribution statement

**Sophie A. Twigger:** Writing – review & editing, Validation, Methodology, Investigation, Formal analysis, Data curation. **Blanca Dominguez:** Methodology, Investigation, Formal analysis, Data curation. **Vanesa Porto:** Methodology, Investigation, Formal analysis, Data curation. **Lina Hacker:** Writing – review & editing, Formal analysis, Data curation. **Anthony J. Chalmers:** Conceptualization. **Ross Breckenridge:** Conceptualization. **Martin Treder:** Conceptualization. **Adam C. Sedgwick:** Methodology, Investigation, Formal analysis, Data curation. **Fernando Dominguez:** Writing – review & editing, Supervision, Funding acquisition, Formal analysis, Conceptualization. **Ester M. Hammond:** Writing – review & editing, Writing – original draft, Supervision, Project administration, Funding acquisition, Formal analysis, Conceptualization.

## Declaration of competing interest

RB is the CEO and Board Director of Arjuna Therapeutics. MT is the CSO of Arjuna Therapeutics. FD is scientific advisor and shareholder of Arjuna Therapeutics and has patents on Ag5 synthesis and the therapeutic applications.

## Data Availability

No data was used for the research described in the article.
