# Supplementary material for: The activity of therapeutic molecular cluster Ag5 is dependent on oxygen level and HIF-1 mediated signalling
Source: Redox Biol. 2024 Aug 22;76:103326. doi: 10.1016/j.redox.2024.103326 (PMC11388176; doi:10.1016/j.redox.2024.103326)

**The activity of therapeutic molecular cluster Ag5 is dependent on oxygen level and HIF-1 mediated signalling**

Sophie A. Twigger^a^, Blanca Dominguez^b^, Vanesa Porto^b^, Lina Hacker^a^, Anthony J. Chalmers^c^, Ross Breckenridge^d^, Martin Treder^d^, Adam C. Sedgwick^e^, Fernando Dominguez^b^ and Ester M. Hammond^a*^

^a^Department of Oncology, University of Oxford, Oxford, OX3 7DQ, UK, ^b^Department of physiology and CIMUS Universidade de Santiago de Compostela, Spain, ^c^School of Cancer Sciences, University of Glasgow, UK, ^d^Arjuna Therapeutics, Milladoiro, Spain, ^e^Department of Chemistry, King's College London, London, SE1 1DB, UK

*Corresponding author

ester.hammond@oncology.ox.ac.uk

**Contents list**

SI Table page 2

SI Figure legends page 2

Chemistry experimental page 4

**SI Tables**

Table S1. **Mutational status of the cell lines used in this study.** Details of cell lines used in this study and their mutational status for key genes involved in tumorigenesis. Wild type is shown in green and mutations orange, with grey showing data not available on the DepMap database. OSCC=oesophageal squamous cell carcinoma, OAC=oesophageal adenocarcinoma, NSCLC=non-small cell lung cancer, CCC=clear cell carcinoma, CAC=colorectal adenocarcinoma. Data collected from DepMap (OmicsSomaticMutations database).

| Cell line | Tumour type | p53 | ARID1A | NFE2L2 | KEAP1 | K-Ras |
| --- | --- | --- | --- | --- | --- | --- |
| OE21 | OSCC | S90F and W91GfsTer32 |  | G81S and D318H |  |  |
| FLO1 | OAC | C277F |  |  |  |  |
| OE33 | OAC | C135Y |  |  |  |  |
| SKGT4 | OAC | Q100Ter |  |  |  | G12A |
| A549 | NSCLC |  |  |  | G333C | G12S |
| H460 | NSCLC |  | I2135_L2136del |  | D236H | Q61H |
| RCC4 | CCC |  |  |  |  |  |
| RKO | CAC |  | D423TfsTer2, T939RfsTer2, D1850TfsTer33 and P1115QfsTer46 |  | G350S |  |

**SI Figure legends**

Figure S1. **Synthesis and validation of novel GSH probe FL-1.**

**A.** The proposed reaction of FL-1 with GSH to afford highly fluorescent 3-O-Methylfluorescein.

**B.** UV-Vis spectra of FL-1 (25 µM) with and without the addition of GSH (1 mM) in PBS buffer (pH = 7.40, 1 % DMSO)

**C.** Fluorescence spectra of FL-1 (5 µM) with increasing additions of GSH (250 µM, 500 µM, and 2 mM – 30 min incubation) in PBS buffer (pH = 7.40, 1 % DMSO). λ_ex_ = 480 nm, Slit widths: 10 nm and 2.5 nm.

**D.** Fluorescence fold change of FL-1 (5 µM) with GSH (2 mM – 30 min incubation), Cys (2 mM – 30 min incubation), and Lys (2 mM – 30 min incubation) in PBS buffer (pH = 7.40, 1 % DMSO). λ_ex_ = 480 nm, Slit widths: 10 nm and 2.5 nm.

**E**. A549 cells were treated with FL-1 (10 μM, 1 h), followed by fluorescence measurement in a POLARstar plate reader. NEM (200 μM, 20 min) and NAC (2 mM, 5 h) were used as controls to ensure the signal measured was due to the presence of GSH.

Data shown in E is n=3. Black dots on the graphs shown represent biological repeats (each of which was carried out in triplicate). Data presented as mean + SEM. Statistical testing was done using an unpaired t-test. ***p<0.001.

Figure S2. **Hypoxic cells are less sensitive to Ag5.**

**A**. A549 cells were exposed to the indicated oxygen concentrations for 6 h. Where indicated Ag5 (1 μM) was added for 1 h prior to harvesting. Western blotting was then carried out for HIF-1α, p53-S15, p53 and β-actin as a loading control.

**B**. Schematic representation of cell viability assays with Ag5 with pre-exposure to hypoxic conditions. Cells were placed in hypoxia for 6 h and then treated with Ag5 for 1 h.

**C**. H460 cells were exposed to 21, 2, 0.5 and <0.1 % O_2_ (6 h) and then treated with Ag5 (0, 0.5, 1, 1.25, 1.5 µM) for 1 h, followed by an MTT assay 20 h later.

**D**. Schematic representation of cell viability assays with Ag5 with no pre-exposure to hypoxic conditions. Cells were exposed to Ag5 for 1 h.

**E**. H460 cells were treated with Ag5 (0, 0.5, 1, 1.25, 1.5 μM) and then immediately exposed to 21, 2, 0.5 and <0.1 % O_2_. Media was changed after 1 h and an MTT assay was carried out 20 h later.

**F**. H460 cells were exposed to 21 and 2 % O_2_ (4 h) and then treated with Ag5 (0, 1, 2, 3 μM) for 15 min, followed by western blotting for oxidised and reduced PRDX3, with GAPDH as a loading control.

**G**. Quantification of the percentage of oxidised/reduced PRDX3 in H460 cells after exposure to 21 and 2 % O_2_ (4 h) followed by Ag5 treatment (15 min) and western blotting.

Data shown in C, E, F, and G are n=3/4. Data shown in A is n=2. Black dots on the graphs shown represent biological repeats (each of which was carried out in triplicate). Data presented as mean + SEM. Statistical testing was done using a two-way ANOVA test with each bar compared to the 21 % O_2_ counterpart. *p<0.05, **p<0.01, ***p<0.001, ns= non-significant.

Figure S3. **Impact of hypoxia on sensitivity to Ag5 and radiation.**

**A.** RKO cells were exposed to 21 and 2 % O_2_ (6 h) and then treated with Ag5 (0, 0.5, 0.75, 1, 1.25 µM) for 1 h, followed by an MTT assay 20 h later.

**B.** RKO and RKO^HIF-/-^ cells were exposed to the O_2_ levels shown (6 h) and GSH levels were determined (GSH GLO assay). In each case NEM (200 μM, 6 h) was included to verify that the luminescence recorded was due to GSH.

**C.** Schematic representation of HIF-1 mediated signalling to PDH.

**D.** A549 cells were exposed to 21 or <0.1 % O_2_  (5 h) and then exposed to radiation (0, 2, 4 Gy) without reoxygenation of the hypoxic (<0.1% O_2_) cells, followed by a colony survival assay.

Data shown in A and D are n=3/5. Data shown in B is n=1. Black dots on the graphs shown represent biological repeats (each of which was carried out in triplicate). Data presented as mean + SEM. Statistical testing was done using an unpaired t-test. *p<0.05, **p<0.01, ****p<0.0001, ns= non-significant.

**Experimental
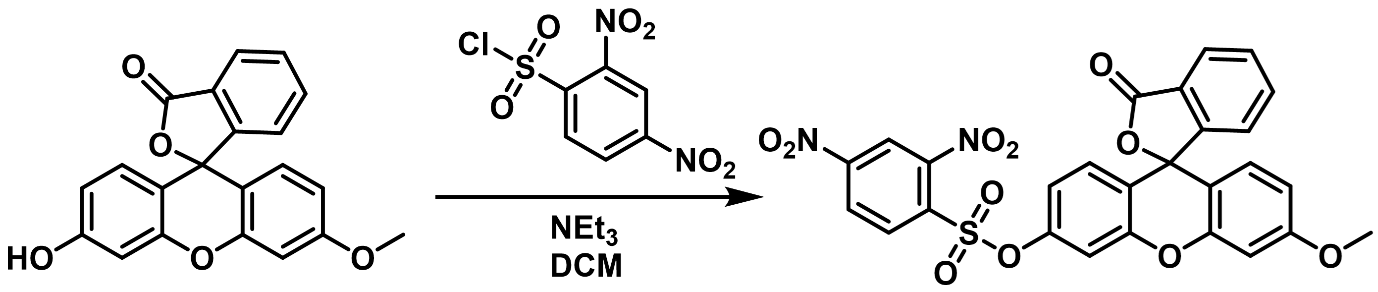
- Synthetic Procedure of FL-1**

Scheme 1. **Synthesis of FL-1 from 3-O-Methylfluorescein**

3'-methoxy-3-oxo-3H-spiro[isobenzofuran-1,9'-xanthen]-6'-yl 2,4-dinitrobenzenesulfonate (FL-1)

2,4-Dinitrobenzenesulfonylchloride (0.288 g, 1.08 mmol) in DCM (5 mL) was added to 3-O-methylfluorescein (0.250 g, 0.72 mmol) and NEt_3_ (1.46 mL, 1.08 mmol) in DCM (10 mL) at 0 ^o^C. The reaction was monitored via TLC chromatography. Once the reaction was deemed complete, H_2_O (15 mL) was added, and the organic layer was removed and washed with H_2_O (3 x 25 mL), brine (1 x 25 mL), dried with MgSO_4_ and concentrated *in vacuo*. The crude material was purified via column chromatography (EtOAc /Hexane – 25/75) to afford the title compound as a white solid (0.175 g , 0.30 mmol, 42 %). ^1^H NMR (400 MHz, CDCl_3_) δ 8.67 (d, *J* = 2.2 Hz, 1H), 8.53 (dd, *J* = 8.6, 2.2 Hz, 1H), 8.26 (d, *J* = 8.6 Hz, 1H), 8.06 – 7.99 (m, 1H), 7.67 (m, 2H), 7.21 (d, *J* = 2.4 Hz, 1H), 7.15 (dt, *J* = 7.6, 1.0 Hz, 1H), 6.90 (dd, *J* = 8.7, 2.4 Hz, 1H), 6.81 (d, *J* = 8.7 Hz, 1H), 6.77 (d, *J* = 2.4 Hz, 1H), 6.72 – 6.62 (m, 2H), 3.84 (s, 3H); ^13^C NMR (101 MHz, CDCl_3_) δ 168.99, 161.68, 152.59, 152.12, 151.92, 151.09, 149.43, 149.03, 135.37, 134.06, 133.31, 130.17, 130.06, 129.03, 126.68, 126.35, 125.32, 123.88, 120.48, 119.50, 117.23, 112.42, 110.94, 110.52, 100.92, 81.79, 55.67. HRMS: m/z calculated for C_27_H_16_N_2_O_11_S [M+H]^+^ 577.0548, found 577.0544.

**NMR Spectra**

**3'-methoxy-3-oxo-3H-spiro[isobenzofuran-1,9'-xanthen]-6'-yl 2,4-dinitrobenzenesulfonate (FL-1) –** ^1^H NMR (400 MHz, CDCl_3_)


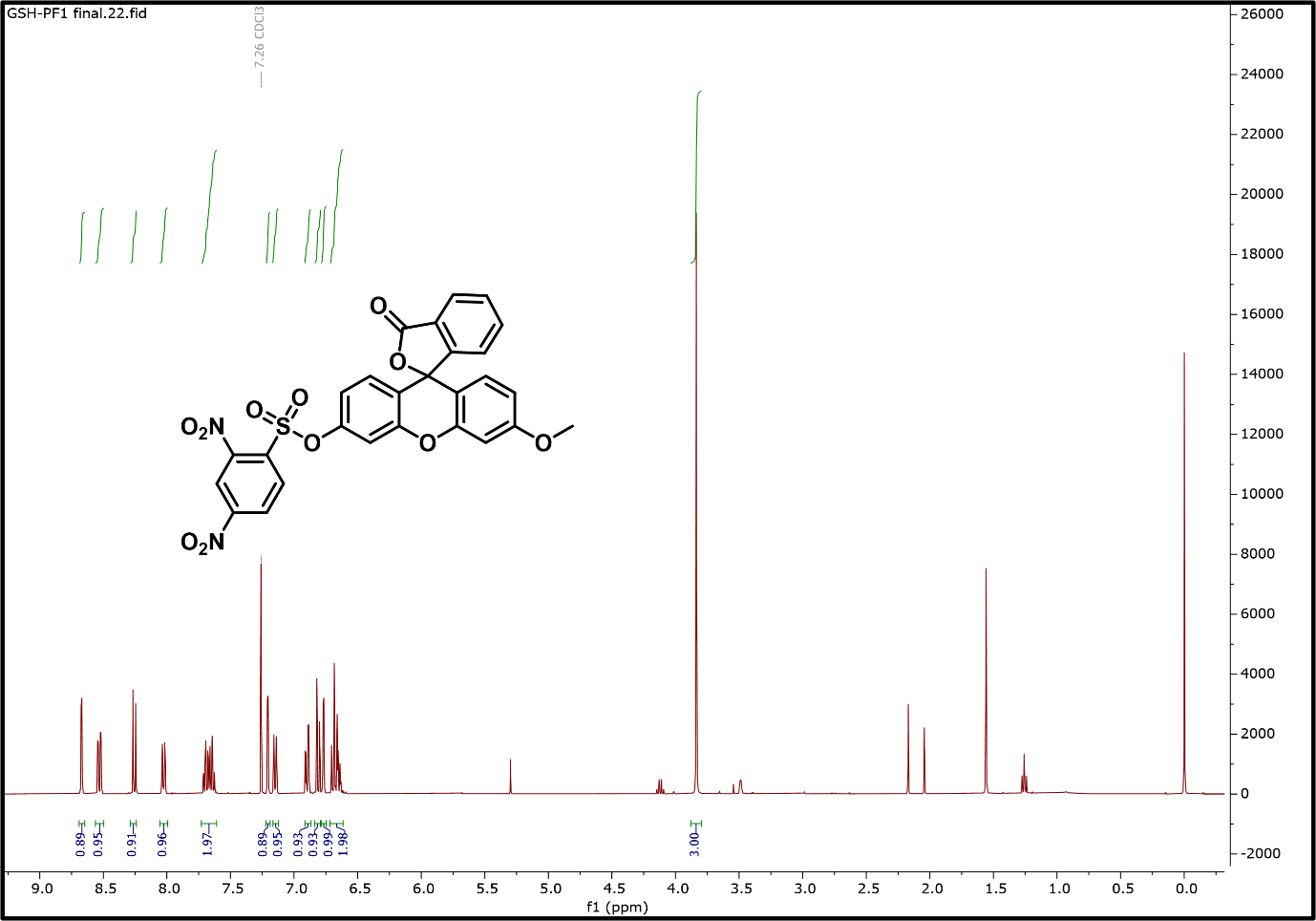


**3'-methoxy-3-oxo-3H-spiro[isobenzofuran-1,9'-xanthen]-6'-yl 2,4-dinitrobenzenesulfonate (FL-1) –** ^13^C NMR (101 MHz, CDCl_3_)


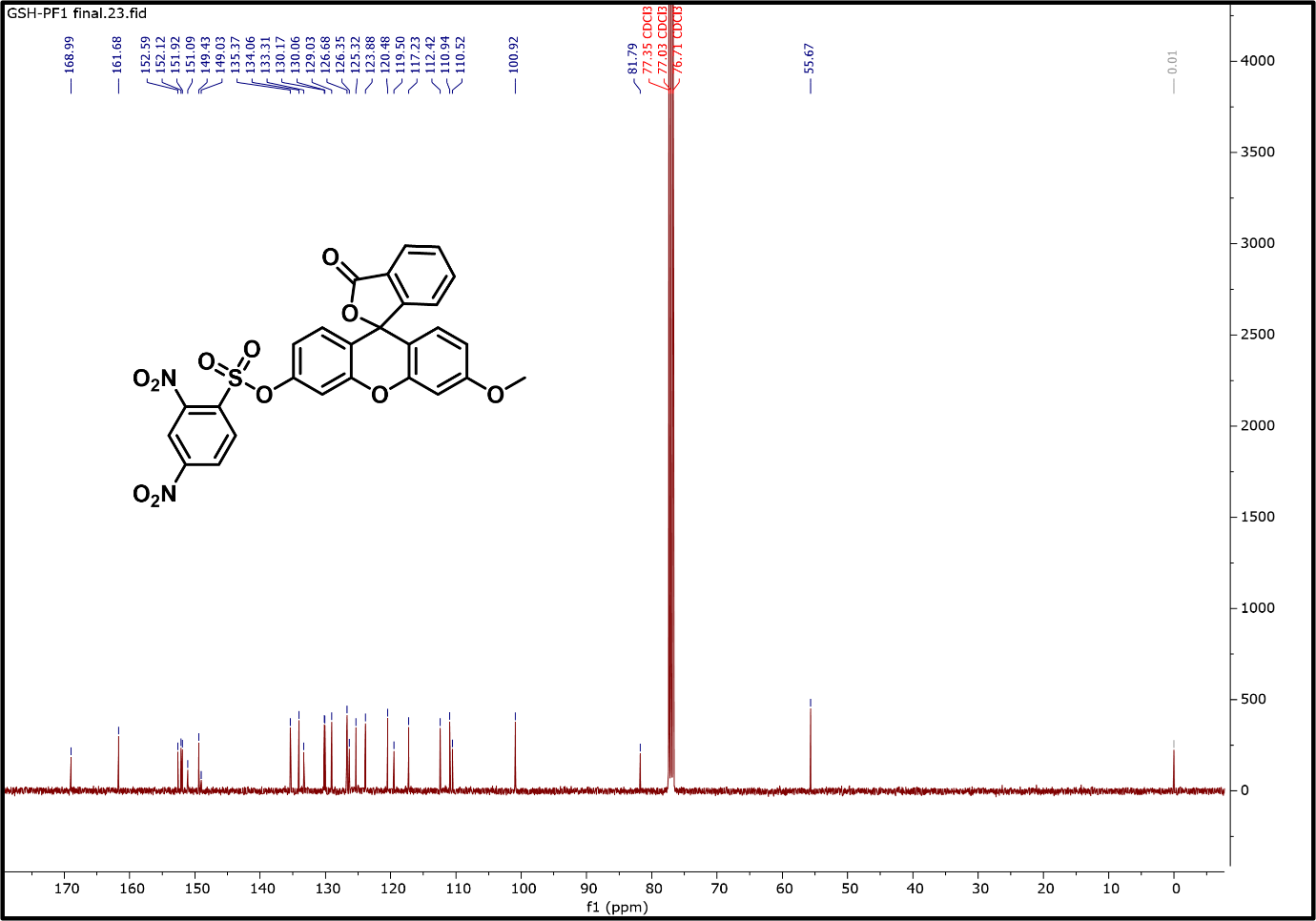

Supplement: Multimedia component 1 [file mmc1.docx]
