# Supplementary material for: The activity of therapeutic molecular cluster Ag5 is dependent on oxygen level and HIF-1 mediated signalling
Source: Redox Biol. 2024 Aug 22;76:103326. doi: 10.1016/j.redox.2024.103326 (PMC11388176; doi:10.1016/j.redox.2024.103326)
Supplement: Multimedia component 2 [file mmc2.pptx]

## Slide 1
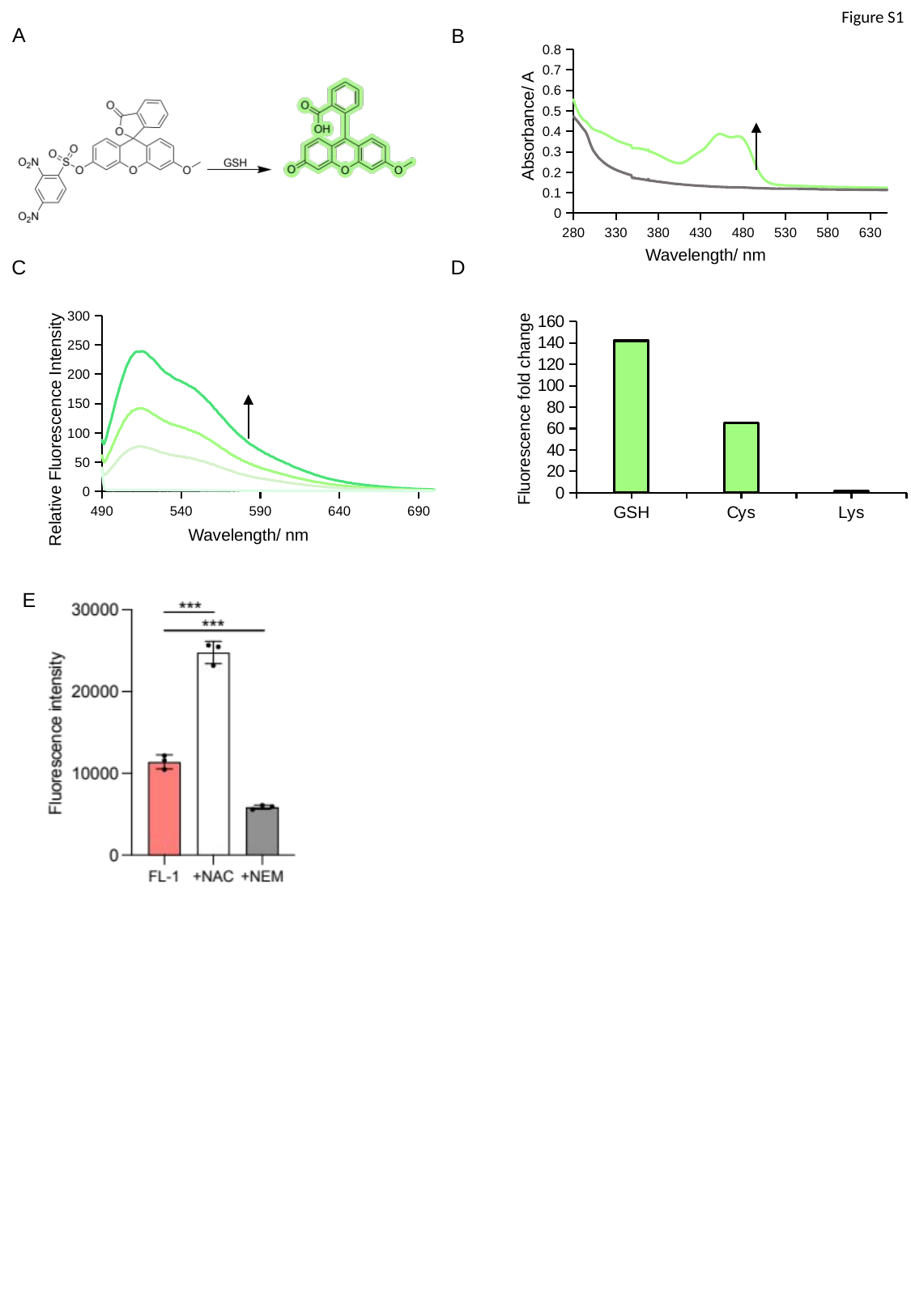

Figure S1
A
B
### Chart
| Category | | |
|---|---|---|Absorbance/ A
Wavelength/ nm
C
D
### Chart
| Category | |
|---|---|
| GSH | 142.0 |
| Cys | 65.0 |
| Lys | 1.64 |Fluorescence fold change
### Chart
| Category | | | | |
|---|---|---|---|---|Relative Fluorescence Intensity
Wavelength/ nm
E

## Slide 2
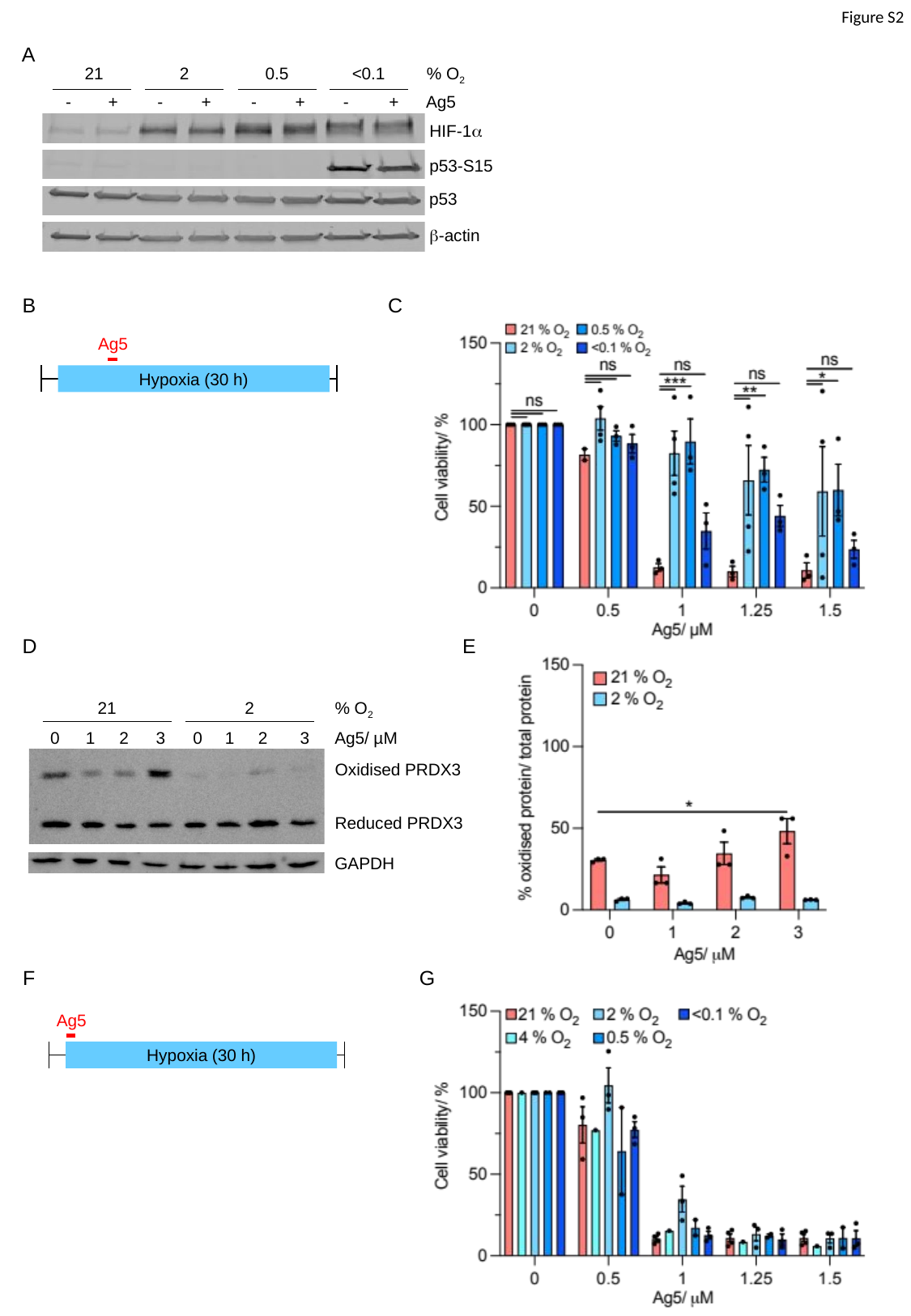

Figure S2
A
21
2
0.5
<0.1
% O2
-
+
-
+
-
+
-
+
Ag5
HIF-1a
p53-S15
p53
b-actin
C
B
Ag5
Hypoxia (30 h)
D
E
21
2
% O2
0
1
2
3
0
1
2
3
Ag5/ µM
Oxidised PRDX3
Reduced PRDX3
GAPDH
F
G
Ag5
Hypoxia (30 h)

## Slide 3
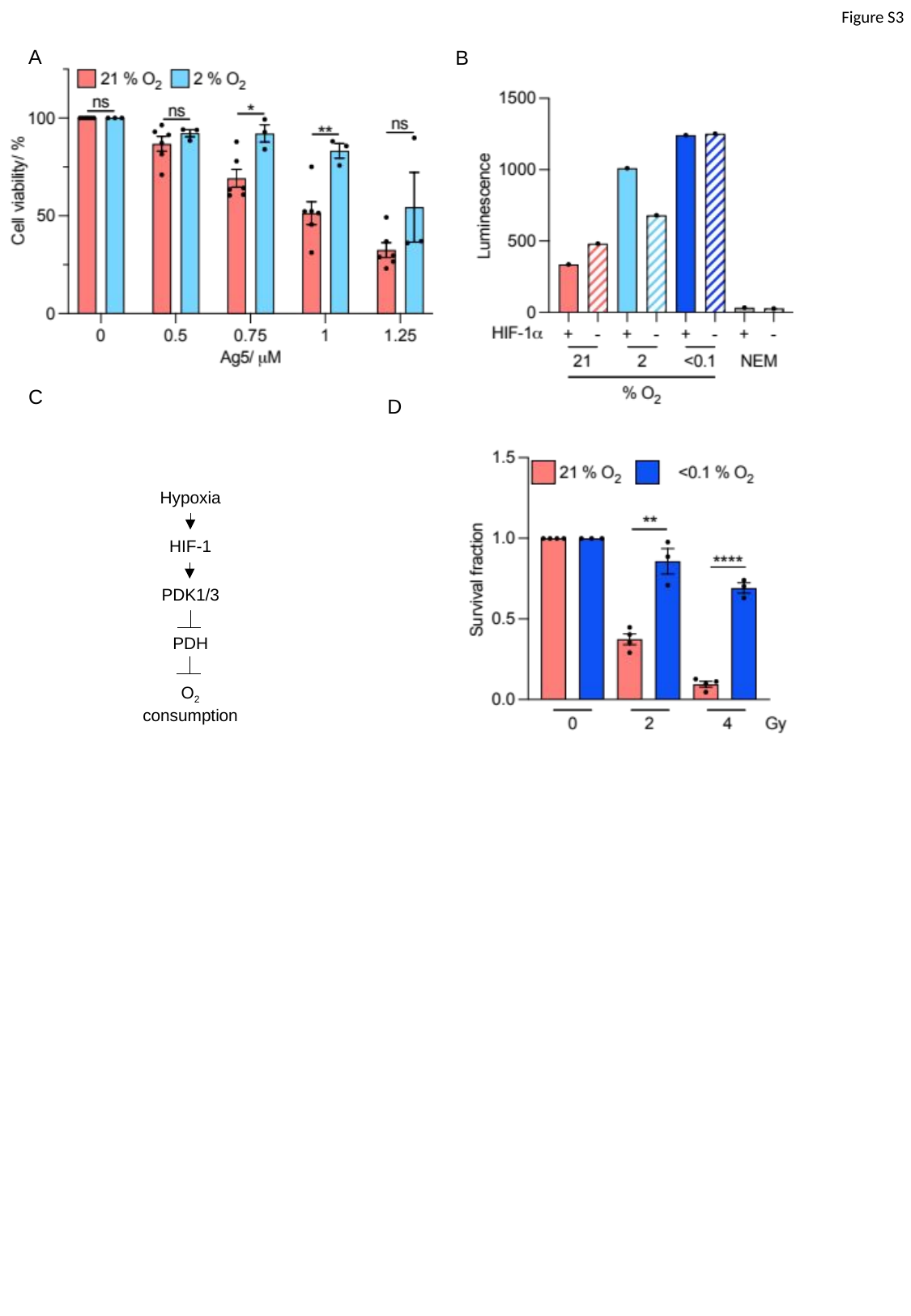

Figure S3
A
B
C
D
Hypoxia
HIF-1
PDK1/3
PDH
O2 consumption
